# Editorial: Preserving emotional health in aging: unraveling the neural mechanisms and implications for neurodegenerative diseases

**DOI:** 10.3389/fnagi.2025.1772872

**Published:** 2026-01-23

**Authors:** Quelen Iane Garlet, Daniel Felsky, Kiyotaka Nemoto

**Affiliations:** 1 Behavioral Neuroscience Lab, Pharmacology Department, Federal University of Paraná, Curitiba, Brazil; 2 Krembil Centre for Neuroinformatics (KCNI), Toronto, ON, Canada; 3 Centre for Addiction and Mental Health (CAMH), Toronto, ON, Canada; 4 Department of Medical Informatics and Management and Psychiatry, Institute of Medicine, University of Tsukuba, Tsukuba, Japan

**Keywords:** cognitive decline, elderly population, emotional health, global mental health, neuroimage

Our behavior throughout life is shaped by how we interact with the world, influenced by how we perceive our environment and express emotions. As we age, experiences refine our cognitive model through brain learning processes (Privitera et al., 2024; Gooijers et al., 2024). However, how variations in molecular and neural mechanisms across trajectories of healthy aging and cognitive decline influence emotional expression remains unclear. This aspect of emotional and cognitive resilience is crucial for maintaining daily functioning in later life. The demographic increase of older adults is accompanied by a rise in neurodegenerative and behavioral disorders, requiring specific approaches to health in advanced adult life (Stancu et al., 2025). Cognition is not an isolated construct but a product of emotional, social, psychological, and biological interplay. This Research Topic brings together interdisciplinary studies addressing emotional, cognitive, and neural factors shaping late-life resilience, integrating individuals' experiences with contextual and physiological factors modulating cognition and wellbeing.

## Protective psychological and lifestyle factors

Day-to-day functioning in late life depends on preserving cognition and emotional health. Emotional resilience emerges as a cornerstone for longevity, requiring synergy among modifiable factors such as physical activity, social networks, education, and psychological wellbeing. Self-reported hopefulness is positively associated with healthy cognitive function in older adults, with this protection being conditional: the association is significantly stronger in individuals with moderate to high physical activity levels (B = 7.409, *p* < 0.001) (Lee et al.). This suggests psychological and lifestyle benefits are synergistic, potentially improving neuroplasticity through increased brain-derived neurotrophic factor (BDNF) and enhanced cortical-limbic connectivity. The presence of social support networks also predicts cognitive strength, serving as a negative predictor for cognitive frailty (B = −0.066, *p* < 0.01), with psychological resilience mediating this effect (Li et al.). Education plays a crucial role in this association, with higher educational attainment serving as a protective factor against cognitive frailty. Interestingly, the positive effect of social support is progressively weakened as educational level increases, suggesting that social support is particularly crucial for improving psychological resilience in individuals facing educational vulnerability.

## Risk factors and their interplay

Management of risk factors for neurocognitive disorders represents another major aspect of health in older adults. Mental health challenges and chronic pain work synergistically toward cognitive decline, demanding expedient management. A longitudinal association exists between chronic multi-site pain and rapid cognitive decline (adjusted OR = 1.30, 95% CI: 1.14–1.48), with depressive symptoms serving as a significant predictor (adjusted OR = 1.49, 95% CI: 1.32–1.68). Depression mediates the effect of pain on cognitive decline, accounting for approximately 25.71% of the total effect (Cheng et al.). When sleep disturbances and hypertension are added to this equation, the impact becomes even more pronounced. Hypertensive older adults experience cognitive decline linearly associated with depression (OR = 1.5) and non-linearly (U-shaped curve) associated with sleep duration (Fan et al.). Both short sleep duration (< 6.6 h) and long sleep duration (>7.7 h) negatively impact cognition, with an inflection point at 7.3 h. This U-shaped effect is more pronounced in individuals with higher education, suggesting that although education provides protection through cognitive reserve, highly educated individuals may face higher risks of sleep schedule disturbances.

## Global mental health disparities

Both cognitive decline and mental health impairments co-occur in older adults and potentiate each other. Elderly psychiatric symptoms are significantly impacted by sociodemographic factors. In regions with low sociodemographic indices, mental health is particularly impaired, and women bear a disproportionate burden (Chen et al.). The age-standardized disability-adjusted life years (DALY) rate was highest in low-SDI regions (2,424 per 100,000) compared to high-SDI regions (1,934 per 100,000). Females showed higher age-standardized incidence rates (7,484 vs. 5,182 per 100,000) and DALY rates (2,198 vs. 1,760 per 100,000) than males. Projections extending to 2035 indicate an increase in the standardized DALY rate, highlighting that public health strategies should prioritize widening access to mental healthcare with sex-specific interventions.

## Neural mechanisms: gaps and opportunities

Despite advances in neuroimaging technology for assessing Central Nervous System disorders, the fundamental neurobiological basis of psychiatric symptoms in neurodegenerative diseases remains poorly understood. While diagnostic values and cognitive symptoms in dementia have seen significant progress, neuroimaging findings of neuropsychiatric symptoms are less consistent, particularly regarding affected brain regions (Sone and Shinagawa). These inconsistencies may arise in neuroimaging modalities, analytical methods, temporal variability of symptoms, and subjective assessments. Recent findings propose that abnormalities in temporal and frontal lobes (particularly the prefrontal cortex) and limbic structures such as the cingulate gyrus, amygdala, and hippocampus play roles in cognitive decline. However, morphological changes are better detected in advanced disease stages. New methods capable of evaluating connectivity among these implicated areas are necessary to detect patterns of connectivity changes in early stages of cognitive decline. Artificial intelligence is emerging as a powerful tool, providing means to perform multimodal neuroimaging and deep neural network analysis to help predict cognitive decline based on neuroimaging together with symptoms, patient sociodemographic characteristics, and lifestyle factors.

## Conclusion

This Research Topic highlights the multifaceted nature of emotional and cognitive resilience in aging. Preserving emotional health in older adults requires a comprehensive approach addressing protective factors (hopefulness, social support, education, physical activity) while managing risk factors (pain, depression, sleep disturbances, cardiovascular conditions). The synergistic effects of these factors underscore the need for integrated interventions promoting both psychological wellbeing and physical health. Persistent global disparities in mental health burden emphasize the urgency of equity-focused public health strategies, particularly for vulnerable populations in low-resource settings and for women. Bridging the gap between advanced neuroimaging technologies and our understanding of neurobiological substrates of emotional dysfunction represents a critical frontier. Future research incorporating multimodal approaches and artificial intelligence holds promise for early detection and targeted interventions, supporting healthy cognitive and emotional aging for all.
